# Consensus Statement on Drug-Coated Balloons in Coronary Artery Disease from the Cardiovascular Intervention Association of Thailand

**DOI:** 10.3390/jcm14217505

**Published:** 2025-10-23

**Authors:** Pannipa Suwannasom, Korakoth Towashiraporn, Worawut Roongsangmanoon, Wiwat Kanjanarutjawiwat, Purich Surunchupakorn, Muenpetch Muenkaew, Ply Chichareon, Pisit Hutayanon, Anek Kanoksilp, Mann Chandavimol

**Affiliations:** 1 Division of Cardiology, Department of Internal Medicine, Faculty of Medicine, Chiang Mai University, Chiang Mai 50200, Thailand; pannipa_100@hotmail.com; 2 Her Majesty Cardiac Center, Faculty of Medicine, Siriraj Hospital, Mahidol University, Bangkok 10700, Thailand; korakonov@yahoo.com; 3 Division of Cardiology, Department of Internal Medicine, Faculty of Medicine, Srinakharinwirot University, Nakhon Nayok 26120, Thailand; jrworawut@yahoo.co.th; 4 King Prajadhipok Memorial Hospital, Chanthaburi 22000, Thailand; mdwiwat@gmail.com; 5 Central Chest Institute of Thailand, Nonthaburi 11000, Thailand; winnermd57@gmail.com (P.S.); akanok@gmail.com (A.K.); 6 Division of Cardiology, Department of Internal Medicine, Faculty of Medicine, Thammasat University, Pathum Thani 10120, Thailand; muenpetch@gmail.com (M.M.); dr_pisit@yahoo.com (P.H.); 7 Division of Cardiology, Department of Internal Medicine, Faculty of Medicine, Prince of Songkla University, Songkhla 90110, Thailand; plychi83@gmail.com; 8 Division of Cardiology, Department of Medicine, Faculty of Medicine, Ramathibodi Hospital, Mahidol University, Bangkok 10400, Thailand

**Keywords:** drug-coated balloon, coronary artery disease, intravascular imaging, small vessel disease, dual antiplatelet therapy

## Abstract

**Background**: Drug-coated balloons (DCBs) have transformed percutaneous coronary intervention (PCI) by delivering antiproliferative drugs without leaving a permanent scaffold. DCB is initially indicated for in-stent restenosis (ISR) and now has expanded indication for treating small vessel disease and bifurcation lesions. However, there is a heterogeneity in the patient and lesion selection, lesion preparation techniques, and the optimal duration of dual antiplatelet therapy after DCB angioplasty. The Cardiovascular Intervention Association of Thailand (CIAT) developed a consensus statement on DCB use in coronary interventions. **Methods**: The CIAT expert panel systematically reviewed randomized controlled trials, meta-analyses, and real-world studies evaluating DCB therapy. Procedural strategies, imaging guidance, physiologic assessment, and antiplatelet therapy protocols were appraised. The recommendations were developed and put to an online vote. Consensus was defined when the recommendation reached 80% of votes in support of “agree” or “neutral”. **Results**: Clinical evidence demonstrates that DCBs achieve comparable outcomes to drug-eluting stents (DESs) in selected lesions while enabling shorter durations of dual antiplatelet therapy (DAPT), particularly beneficial for high-bleeding-risk patients. Optimal outcomes require meticulous lesion preparation, appropriate balloon sizing, and controlled vessel dissection. Intravascular imaging and physiologic assessment further refine procedural precision, while hybrid strategies combining DCBs and DESs address complex lesions and multivessel disease. The final document presents 15 consensus statements addressing indications, procedural techniques, imaging and physiologic guidance, and antiplatelet therapy recommendations. **Conclusions**: DCB angioplasty can be an alternative or complement to therapeutic options to DESs across multiple clinical and anatomical scenarios. The CIAT consensus provided structured recommendations to support DCB therapy in contemporary practice.

## 1. Introduction

Drug-eluting stents (DESs) are the mainstay therapy when choosing percutaneous coronary intervention (PCI) as the revascularization method. DESs have demonstrated excellent long-term outcomes in various clinical settings; however, several limitations have precluded complete revascularization for both anatomical and clinical settings. DESs are related to delayed vascular healing, risk of very late stent thrombosis, challenges with future re-access of the vessel, and the need for extended dual antiplatelet therapy (DAPT), which increases bleeding risk.

Drug-coated balloons (DCBs) have emerged as an innovative, stent-free PCI approach. DCBs allow for the local delivery of antiproliferative drugs to the vessel wall without leaving behind a permanent implant. This approach facilitates natural vascular healing, preserves vasomotor function, and enables shorter durations of DAPT in selected patient populations. Despite the initial idea to develop DCBs to treat in-stent restenosis (ISR), DCBs have progressively expanded into broader indications, including de novo small vessel disease, bifurcation lesions, and high-bleeding-risk patients. Over the past decade, growing evidence from randomized clinical trials (RCTs) and real-world studies has supported their efficacy and safety in these settings, positioning DCBs as an alternative or complement to DESs in carefully selected patients. However, substantial heterogeneity persists in clinical practice. To promote standardized best practices and address the variation in practice, the Cardiovascular Intervention Association of Thailand (CIAT) developed an expert consensus panel to provide updated, evidence-based guidance on DCB use in contemporary coronary intervention.

This consensus statement summarizes current clinical evidence and practical considerations, focusing on four key domains: (1) indication for DCB angioplasty, (2) lesion preparation and assessment, (3) deployment technique and adjunctive use of physiology or imaging, and (4) duration of dual antiplatelet after DCB angioplasty.

## 2. Methodology

The CIAT invited an established expert panel to develop evidence-based recommendations on the clinical use of DCBs in coronary artery disease. Eighteen interventional cardiologists with recognized expertise in coronary intervention participated in the consensus process.

A comprehensive literature search was performed in PubMed, including registries, randomized controlled trials, meta-analyses, and relevant review articles pertaining to the indications, procedural techniques, and antiplatelet strategies associated with DCB treatment. Each article was reviewed and appraised for methodological quality and clinical relevance.

The levels of evidence supporting each recommendation were categorized as high, moderate, low, or very low, reflecting the panel’s confidence that the estimated effect is close to the true effect. Draft recommendations were developed and defined during a virtual meeting held in July 2025. The final position statements were subjected to an online voting process. Each recommendation was evaluated using a three-point scale (agree, neutral, or disagree). Consensus was defined as ≥80% of votes indicating “agree” or “neutral”.

## 3. Clinical Evidence on DCBs (Lesion and Clinical Setting)

### 3.1. Lesion

#### 3.1.1. In-Stent Restenosis

In-stent restenosis (ISR) remains a major challenge in treating patients with coronary artery disease undergoing PCI. Several clinical studies have demonstrated the efficacy of DCBs in treating ISR, with favorable angiographic and clinical outcomes observed in both short-term and long-term follow-up. These studies have primarily compared DCBs with balloon angioplasty (BA) and 1st- or 2nd-generation drug-eluting stents (DESs).

The PACCOCATH ISR-I (Treatment of In-Stent Restenosis by Paclitaxel-Coated PTCA Balloons) trial showed that the paclitaxel-coated balloon (PCB) significantly reduced late lumen loss (LLL) (*p* = 0.002) and major adverse cardiac events (MACEs) (4% vs. 31%, *p* = 0.01) compared to balloon angioplasty (BA) alone at 6 months [1]. At 24 months, PCBs also had lower target lesion revascularization (TLR) rates (*p* = 0.001) [2]. The PEPCAD-DES trial (Treatment of DES-In-Stent Restenosis with SeQuent Please Paclitaxel-Eluting PTCA Catheter) confirmed the superiority of PCBs over BA for ISR in DES (ISR-DES) at six months, reducing LLL (*p* < 0.001) and the binary restenosis rate (17.2% vs. 58.1%, *p* < 0.001) [3].

For ISR in bare-metal stents (ISR-BMS), the PEPCAD II trial (Paclitaxel-Eluting PTCA-Balloon Catheter in Coronary Artery Disease II) found PCB superior to paclitaxel-eluting stents (PESs) in reducing LLL (*p* = 0.03), though MACE rates were similar at 12 months (*p* = 0.08) [4]. In contrast, the RIBS V trial (Restenosis Intra-Stent of Bare-Metal Stents: Paclitaxel-Eluting Balloon vs. Everolimus-Eluting Stent) found that everolimus-eluting stents (EESs) outperformed PCBs, with a larger minimal lumen diameter (MLD) (*p* < 0.001) and lower diameter stenosis (*p* < 0.001). Although LLL and binary restenosis did not differ significantly, EESs showed better long-term angiographic outcomes with a lower TLR rate at three years (*p* = 0.04) [5]. Rates of cardiac death (2% vs. 1%), myocardial infarction (4% vs. 5%), and target vessel revascularization (TVR) did not show significant differences [6].

The ISAR-DESIRE 3 trial (Intracoronary Stenting and Angiographic Results—Drug-Eluting Stent In-Stent Restenosis: 3 Treatment Approaches) compared BAs, PCBs, and PESs for ISR-DES. PCBs were non-inferior to PESs for diameter stenosis and superior to BAs (*p* < 0.0001) at 6–8 months [7]. In the long-term follow-up to 10-years, no significant differences in clinical outcomes between PESs and PCBs were demonstrated [8]. Similarly, the PEPCAD China ISR trial (A Prospective, Multicenter, Randomized Trial of Paclitaxel-Coated versus Paclitaxel-Eluting Stents for the Treatment of Drug-Eluting Stent In-Stent Restenosis) found DCBs non-inferior to PESs in reducing LLL at 9 months (*p* for non-inferiority = 0.0005) [9].

The RIBS IV trial (Restenosis Intra-Stent of Drug-Eluting Stents: Drug-Eluting Balloon vs. Everolimus-Eluting Stent) demonstrated that EESs were more effective than DCBs, with greater MLD (*p* < 0.01), net lumen gain (*p* < 0.01), diameter stenosis (*p* < 0.01), and lower incidence of MACEs (*p* = 0.04), driven by a reduced repeat revascularization rate (8% vs. 16%; *p* = 0.035) [10]. At the three-year follow-up, the EESs group continued to show a significantly lower MACE rate (*p* = 0.04) [11]. The DARE trial (Drug-Eluting Balloon for In-Stent Restenosis) compared PCBs and EESs for ISR-BMS (44%) and ISR-DESs (56%). Post-procedure data revealed that EESs had better post-procedural angiographic outcomes, PCBs were non-inferior for MLD at 6 months (*p* for non-inferiority < 0.0001), with no significant difference in TVR at 12 months (EES 7.1% vs. PCB 8.8%; *p* = 0.65) [12]. Table 1 summarizes the landmark studies of DCBs in treating ISR [13,14,15,16,17,18,19]. Due to the limited power of individual trials to assess clinical endpoints, meta-analyses have been conducted to compare the outcomes of DCBs versus DESs for any ISR. The pooled individual patient data from 10 RCTs (DAEDALUS) demonstrated long-term efficacy and safety between DCB angioplasty and repeat DES implantation in patients with BMS-ISR at the 3-year follow-up. However, in DES-ISR, repeat DES implantation provides similar safety to DCB angioplasty but superior efficacy [20]. CIAT statement of DCB for the treatment of ISR as shown in Box 1.

Box 1 **Statement of DCB for the treatment of ISR.**

Statement 1: DCB angioplasty is recommended as a valid alternative to repeat DES implantation for the treatment of ISR, with comparable long-term efficacy and safety, especially in BMS-ISR. In DES-ISR, repeat DES may offer superior efficacy but similar safety compared with DEB.

- Level of Evidence: High.
- Level of consensus: 100% Agree; 0% Neutral; 0% Disagree.

#### 3.1.2. De Novo Lesions

##### Small Vessel Coronary Disease

According to the consensus document of the Drug Coated Balloon Academic Research Consortium (DCB-ARC), small vessel coronary disease (SVD) is defined as coronary lesions in vessels with a diameter less than 2.75 mm [21]. The results of DESs in SVD, as compared to normal vessel size, have demonstrated higher rates of ISR in long-term outcomes. To address this, several RCTs have assessed the feasibility of using DCB in SVD. These studies have compared DCBs with plain old balloon angioplasty (POBA) [22], BMS [23], and DESs [24,25,26,27,28], as summarized in Table 2 [22,24,26,28,29,30]. There were conflicts in the long-term outcomes from the meta-analyses. One has reported that there was little evidence that DESs were superior to DCBs in small vessels [31], whereas another reported that DCBs had a trend toward higher rates of MACEs and MI at the 3-year follow-up [32]. Despite the comparable outcomes of DCBs to DESs in small vessel disease at short-term, DCBs were less cost-effective than DESs at 3 years in terms of quality-adjusted life expectancy [33]. The discrepancies of the long-term results could be the heterogenous follow-up durations that range from 3 to 13 years among the meta-analyses, the number of pool patients, variability in the DES platform, and the type of excipient that varies among trials (iopromide in the BASKET-SMALL 2 trial, dextran in the PICCOLETO-II trial, and shellac in the RESTORE SVD trial). CIAT statement of DCB for the treatment of small vessel disease as shown in Box 2.

Box 2 **Statement of DCB for the treatment of small vessel disease.**

Statement 2: DCBs may be a reasonable alternative to DESs for PCI in small vessel coronary disease.

- Level of Evidence: Moderate.
- Level of consensus: 100% Agree; 0% Neutral; 0% Disagree.

##### Diffuse Lesion

Diffuse disease is defined as a coronary segment > 25 mm with vessel wall irregularities and no clear focal lesion [21]. Diffuse coronary lesions account for 20% of PCI cases, with around 40% of these patients having diabetes mellitus [34,35]. Conventional treatment with DESs often leads to longer stent lengths required, ISR, TLR, and stent thrombosis. DCBs, whether used alone or in combination with DESs, can reduce the overall stent length. However, evidence from non-RCTs indicated that there is no significant difference in MACEs, TLR, TVR, or stent thrombosis between DCBs and DESs over 3 years of follow-up [34,36].

##### Vessel Size ≥ 3.0 mm

There has been limited data on the safety and efficacy data regarding DCBs in large coronary vessels (larger than 3 mm in diameter). However, reports have indicated a low rate of clinical events and acute vessel closure [23,27,37,38,39,40,41] in this population. The REC-CAGEFREE-I [42] trial investigated the efficacy of DCBs in de novo vessel lesions without restrictions on vessel size. Among 2902 enrolled patients, nearly half had lesions in vessels ≥ 3 mm. The trial showed no significant differences between the DCB and 2nd-generation DES groups in cardiac death (2.3% vs. 1.2%; *p* = 0.053) and target vessel myocardial infarction (TVMI) (1.9% vs. 1.6%; *p* = 0.606). However, the TLR rate was significantly higher after DCB treatment (3.1% vs. 1.2%; *p* = 0.002). Meta-analyses showed that there were no significant differences between DCBs and DESs in clinical outcomes [43,44] despite DCBs yielding inferior angiographic results [44]. Ongoing RCTs such as the REVERSE (Randomized Trial of Drug-Coated Balloon Versus Drug-Eluting Stent for Clinical Outcomes in Patients with Large Coronary Artery Disease; NCT05846893) are currently evaluating the role of DCBs in large-caliber vessels. CIAT statement of DCB for the treatment of vessel size ≥ 3.0 mm as shown in Box 3.

Box 3 **Statement of DCB for the treatment of vessel size ≥ 3.0 mm.**

Statement 3: The use of DCBs in large vessels should be limited in selected cases until more RCT evidence is available.

- Level of Evidence: Low.
- Level of consensus: 93.3% Agree; 6.7% Neutral; 0% Disagree.

##### Calcified Lesion

Coronary calcification is associated with both procedural challenges and adverse long-term outcomes following PCI [45,46,47,48]. Managing coronary calcification often requires various plaque modification techniques to facilitate PCI, including non-compliant (NC) balloons, scoring balloons, cutting balloons, rotational/orbital atherectomy, and intravascular lithotripsy [49]. Traditionally, DESs are deployed after plaque modification in calcified coronary lesions. However, stent-related complications remain a frequent concern. Recently, DCBs have emerged as an alternative therapeutic approach following adequate lesion preparation.

Several retrospective studies have compared DCBs and DESs in this situation. These studies showed that there was no statistically significant difference in clinical or angiographic outcomes between the two strategies as outlined in Table 3 [50,51,52,53].

A meta-analysis of five studies involving 1141 patients comparing DCBs or DESs for calcified lesions found no significant differences in MACEs, cardiac death, MI, and TLR. While DCBs showed inferiority of immediate angiographic outcomes compared to DESs, they demonstrated superior LLL at the 12-month follow-up, highlighting their long-term effectiveness in selected patients [54]. CIAT statement of DCB for the treatment of calcified lesions as shown in Box 4.

Box 4 **Statement of DCB for the treatment of calcified lesions.**

Statement 4: DCB use in calcified lesions requires adequate lesion preparation with plaque modification devices. Current evidence remains limited.

- Level of Evidence: Low.
- Level of consensus: 93.3% Agree; 6.7% Neutral; 0% Disagree.

##### Chronic Total Occlusion

Evidence supporting the use of DCBs in the treatment of chronic total occlusion (CTO) remains limited. To date, all available clinical studies are derived from retrospective studies with relatively small patient populations [55,56,57,58]. The findings from clinical studies investigating the use of DCBs in CTO treatment are summarized in Table 4 [55,56,57,58].

A meta-analysis by Zhao et al. [59] included 511 patients across five retrospective studies and demonstrated favorable outcomes for DCB treatment in CTO lesions. The MACE rate was 13%, with MI occurring in 1.2% of cases, cardiac death in 2.2%, TLR in 10.1% of cases, and TVR in 7.1%. A recent meta-analysis comparing DCBs vs. DESs vs. hybrid strategies in both de novo and in-stent CTO lesions showed no significant difference in TLR among the treatment approaches [60]. The ongoing randomized controlled CO-CTO trial (NCT04881812) [61] aims to compare the hybrid approach and complete DES stenting strategy. The primary endpoint of the study is in-segment percentage diameter stenosis at one-year follow-up, assessed by intravascular ultrasound (IVUS) [61].

##### Vulnerable Plaque

Despite advancements in systemic antithrombotic, lipid-lowering, and anti-inflammatory therapies, patients undergoing PCI remain at heightened residual risk for recurrent coronary events. Beyond stent-related complications, nearly half of PCI patients have residual vulnerable plaques, which contribute significantly to the increased risk of recurrent coronary events [62]. The PREVENT trial evaluated the strategy of preventive stenting, using either bioresorbable vascular scaffolds or DESs in patients with non-culprit vulnerable plaques. Preventive PCI along with optimal medical therapy resulted in a reduction in MACEs during long-term follow-up compared to medical therapy alone [63]. Nevertheless, ethical concerns have emerged regarding stent-related complications in functionally insignificant lesions. Other concerns include the potential for procedural complications such as distal embolization, periprocedural MI, and restenosis, especially in lipid-rich vulnerable plaques.

PCBs provide targeted intracoronary pharmacological treatment to the vulnerable plaques. Preclinical studies suggested that PCBs may reduce inflammation and promote local plaque stabilization [64]. The recently published DEBuT-LRP study evaluated this approach in 45 patients presenting with non-ST-segment elevation acute coronary syndrome. Of these, 20 patients (44%) had lesions with a maximum lipid core burden index (maxLCBI_4mm_) ≥ 325 and received additional treatment with PCBs. Preemptive treatment of these non-flow-limiting, non-culprit vulnerable lipid-rich plaques resulted in a significant reduction in maxLCBI_4mm_ without any safety concerns [65]. DCBs may thus offer a safe and effective alternative, enabling local pharmacological treatment of vulnerable plaques without leaving a permanent implant. However, further large-scale RCTs are essential to validate their long-term safety and efficacy, particularly as an adjunct to systemic medical therapy in secondary prevention strategies.

#### 3.1.3. Bifurcation

Coronary bifurcation lesions are complex and demand careful decision-making, focusing on the optimal treatment of both the main branch (MB) and side branch (SB). Stenting the MB in bifurcation lesions can lead to overstretching of the distal vessel and vessel straightening, both of which may result in a shift of the carina into the side branch. PCBs have demonstrated the capacity for late lumen enlargement (LLE), reflected by a rightward shift in the MLD distribution curve [66]. PCB treatment on the MB showed a larger MLD of the SB ostium at follow-up compared to DESs or optimal medical therapy [67]. In the small study using PCBs to the SB, the ostial lumen area assessed by OCT significantly increased by 76.1% between pre-procedure and follow-up at 9 months [68].

To date, hybrid strategy, DCBs in the SB, and DESs in the MB [25] have been used in patients with bifurcation lesions. Previously, it was recommended to apply the DCB to the SB prior to MB stenting to avoid drug loss when crossing the stent strut. Alternatively, DCBs can also be used as part of a provisional stenting strategy to treat the SB. If final kissing balloon angioplasty is needed, conventional balloons are preferred. Some studies have demonstrated that PCBs in the SB, in combination with DESs in the MB, significantly reduce LLL and restenosis in the SB [69,70]. The PEPCAD-BIF randomized trial reported a significant reduction of 9-month angiographic LLL and binary restenosis in the PCB cohort compared to the conventional balloon angioplasty cohort for the SB treatment [71]. Recently, the DCB-BIF trial compared DCBs to balloon angioplasty for treating side branches after systemic main branch stenting. At the 1-year follow-up, the primary endpoint of cardiac death, TVMI, or clinically driven TLR, was lower in the DCB group compared to the balloon angioplasty group [72]. The SPACIOUS trial indicated that sirolimus DCBs were non-inferior to paclitaxel DCBs in treating de novo non-left main bifurcation lesions after stenting in the main branch with DESs [73]. In left main (LM) bifurcation lesions, this hybrid approach has proven superior to DES-only strategies, including provisional and two-stent techniques, achieving improved LLL and TLF [74,75].

A DCB-only, or “puristic” approach for the treatment of bifurcation lesions is also feasible, particularly for MEDINA 0.1.1 lesions, with or without additional DCB application to the SB. The standard sequence typically involves DCB inflation in the SB first, followed by the MB, without the use of kissing balloon inflation when carina shift is minimal. Carina shift can be minimized by using a balloon-to-artery ratio between 0.8 and 1.0. Although some operators have performed DCB-based kissing balloon inflation, this technique should be used with caution due to the prolonged DCB delivery time and the potential for mechanical interaction between the kissing balloons and the proximal MB [21]. The PEPCAD-V observational study demonstrated promising results using PCBs for MB and SB lesions in combination with BMS in the MB [76]. A recent meta-analysis confirmed the safety of DCBs for MB treatment, with superior SB LLL outcomes compared to DESs or POBA [77]. A recent study of 39 patients with unprotected LM true bifurcation lesions [78] found that DCB therapy had comparable MACEs at the 2-year follow-up versus DESs, suggesting that DCBs may be an alternative therapy in selected patients with adequate lesion preparation.

Finally, the combination of directional coronary atherectomy (DCA) followed by DCBs has emerged as a novel approach for bifurcation lesions, especially in the LM. This strategy reduces the need for stenting and avoids complex stenting techniques, leading to favorable clinical outcomes. It may also help mitigate carina shift in large-vessel bifurcation lesions [79]. CIAT statement of DCB for the treatment of side branches as shown in Box 5.

Box 5 **Statement of DCB for the treatment of side branches.**

Statement 5: PCB angioplasty of side branches in bifurcation lesions is feasible; however, DESs remain standard for main vessel stenting.

- Level of Evidence: Moderate.
- Level of consensus: 86.7% Agree; 13.3% Neutral; 0% Disagree.

### 3.2. Clinical Setting

#### 3.2.1. Acute Coronary Syndrome

PCI in the acute coronary syndrome (ACS) setting carries significant risks of morbidity and mortality during both the index procedure and long-term follow-up [80,81,82,83]. These risks stem from several factors, including thrombus burden, risk of no-reflow, and vasoconstriction affecting stent sizing and a high prevalence of unstable clinical conditions. In particular, patients at high bleeding risk or those requiring early discontinuation of dual antiplatelet therapy (DAPT) are more vulnerable to stent-related complications such as stent thrombosis [84]. DCBs have emerged as a promising alternative for treating ACS. Unlike DESs, DCBs do not rely on permanent implants, reducing the risk of long-term stent-related complications. Moreover, they allow for shorter DAPT durations and are not constrained by vessel sizing issues.

A propensity score matching retrospective study involving 1139 patients with STEMI to compare outcomes between PCBs (*n* = 452) and 2nd-generation DESs (*n* = 687) showed that no significant differences were observed in all-cause mortality and net adverse cardiac events between the two groups up to the 3-year follow-up [85]. Similarly, a prespecified subgroup analysis of the BASKET-SMALL 2 trial (Basel Kosten Effektivitäts Trial Drug-Coated Balloons Versus Drug-Eluting Stents in Small Vessel Interventions) evaluated 214 patients with ACS (STEMI 7%, NSTEMI 50.9%, and UA 42.1%) and showed no significant difference in MACEs between the DCB and DES groups over a 3-year follow-up (hazard ratio 0.71 [95% confidence interval (CI), 0.35–1.45]) [86]. A meta-analysis also found no significant difference in the incidence of MACEs between the two treatments (odds ratio = 0.89, 95% CI [0.57–1.40], *p* = 0.63) [87].

In summary, the clinical evidence supporting the use of DCBs in ACS remains limited. Current data are primarily derived from retrospective studies, small-scale RCTs, and subgroup analyses within larger trials. Therefore, large-scale RCTs are needed to establish the safety and efficacy of DCBs in ACS patients. CIAT statement of DCB for the treatment in ACS patients as shown in Box 6.

Box 6 **Statement of DCB for the treatment in ACS patients.**

Statement 6: Routine use of DCBs in ACS is not recommended outside of selected cases, as current evidence remains limited.

- Level of Evidence: Low.
- Level of consensus: 93.3% Agree; 6.7% Neutral; 0% Disagree.

#### 3.2.2. High Bleeding Risk

The concept of HBR has gained increasing attention in interventional cardiology. In Thailand, up to 38% of PCI patients met HBR criteria, and these individuals experienced nearly a 4-fold increase in mortality [88]. The increasing numbers of PCI in these patients are challenging, particularly regarding the duration of DAPT. Bleeding complications following PCI are associated with increased one-year mortality and adverse outcomes [89,90,91]. DCBs offer benefits over DESs in HBR patients due to their ability to abbreviate DAPT duration or eliminate the need for DAPT. The DEBUT was the first RCT testing DCB vs. bare-metal stent in HBR patients and showed that PCI with DCBs was superior to bare-metal stent in reducing MACEs with the absolute risk difference of −13.2 percentage points [23]. In a subgroup analysis of the HBR patients in the BASKET-SMALL 2 trial [92], DCBs showed similar safety and efficacy as 2nd-generation DESs, and there was a trend toward a reduction in severe bleeding events at 3 years. CIAT statement of DCB for the treatment in HBR patients as shown in Box 7.

Box 7 **Statement of DCB for the treatment in high bleeding risk patients.**

Statement 7: DCB angioplasty is an alternative option in HBR patients, so it can allow for shorter DAPT duration.

- Level of Evidence: Moderate.
- Level of consensus: 93.3% Agree; 6.7% Neutral; 0% Disagree.

#### 3.2.3. Multivessel Coronary Artery Disease

Extensive stenting with multiple long metallic implants can impair vascular physiology, particularly vasomotion, and is associated with higher rates of target vessel failure (TVF). DCB treatment facilitates positive vessel remodeling and offers an appealing approach that minimizes permanent metal implantation. While dedicated studies on a DCB-only strategy in multivessel disease are limited, favorable outcomes have been observed in patients with diffuse or multivessel disease included in pivotal DCB trials [30,93].

A recent retrospective study compared 254 patients with MVD treated with DCBs alone or in combination with DESs against propensity-matched patients treated with 2nd-generation DESs. The hybrid approach achieved a 65.4% reduction in the number of stents and a 63.7% decrease in total stent length. At the 2-year follow-up, the DCB group showed a significantly lower incidence of MACEs compared to the DES group (3.9% vs. 11.0%; *p* = 0.002) [94]. Subgroup analysis showed that the DCB-based approach significantly reduced MACEs in diabetic patients compared to DESs (HR: 0.19; *p* = 0.003), an effect not observed in non-diabetic patients (HR: 0.52; *p* = 0.167) [95].

Overall, DCBs and DESs should be considered complementary rather than competing technologies. The reduction in metal burden in complex multivessel disease represents a compelling strategy, and hybrid approaches integrating DCBs are increasingly preferred in clinical practice.

## 4. DCB Technology

### 4.1. DCB Component (Antiproliferative and Excipient)

DCBs comprise three key components: the balloon, antiproliferative drug, and excipient. Most DCBs utilize semi-compliant balloons to ensure uniform vessel wall contact during inflation. The majority of commercially available DCBs were paclitaxel-based [30,42,96,97,98], although sirolimus [99,100] and its analogs (e.g., biolimus [101,102,103,104], everolimus [105]) have emerged as alternatives.

Paclitaxel exerts its cytotoxic effect during the M phase of the cell cycle, allowing for rapid tissue uptake and prolonged retention, even with brief contact times. In contrast, sirolimus and its analogs act cytostatically during the G1 phase and offer advantages such as a wider therapeutic window, anti-inflammatory properties, and lower restenosis rates. Biolimus BA9 [104], the latest drug in this class, is more lipophilic than sirolimus, enabling improved tissue absorption and retention.

The excipient is one of the important parts of DCBs to control the drug release to the vessel wall in a specific, sufficient, and as short time as possible during the balloon inflation. Ideally the excipient should be hydrophilic to transfer the drug to the vessel wall as well as lipophilic to make the drug have high absorption and retention in vessel intima. The excipients, such as citrate, iopromide, and urea, were used with paclitaxel, whereas phospholipid-based ones, such as polyethylene oxide, were used in -limus-based drugs [96]. The methods used to coat balloons with excipients and anti-proliferative drugs include micropipetting, dipping, spraying, and imprinting and are used to apply drugs to balloons [106]. Micropipetting employs a micropipette to deliver small, controlled volumes of drug solution precisely onto the balloon surface [107], providing high precision and reproducibility with more uniform coatings and consistent drug release [108]. The dipping method immerses the balloon in a drug-containing solution; however, coating uniformity can be challenging, as the solution may not penetrate evenly into the folds of the balloon. The spraying method uses air or atomizers to create a fine mist of drug solution applied to the balloon surface, producing a uniform coating when the balloon is mounted vertically and sprayed from multiple directions [109]. The imprinting method utilizes nanoparticles to bind or imprint the drug onto the balloon surface. Nanoparticles can encapsulate the drug and allow controlled release at the target site, making this technology distinctive among the four coating approaches [110]. An overview of the DCB platforms available in Thailand is presented in Table 5 [30,96,97,98,99,100,102,103,111].

### 4.2. Are All DCBs the Same?

Regarding the class effect of DCBs in CAD treatment, it is important to recognize that not all DCBs are identical. Variations in balloon design, drug type, dosage, excipients, and coating techniques can significantly influence their efficacy and safety profiles. Consequently, a class effect cannot be assumed, and each DCB should be evaluated based on its specific characteristics and clinical trial data. These developments underscore the importance of ongoing research and individualized assessment in optimizing DCB therapy for patients with CAD.

## 5. Technical Aspect for Lesion Preparation

This section will focus on the use of DCBs in treating de novo lesions and in-stent restenosis (ISR) lesions. Lesion preparation is the most important step for the DCB treatment. The key technique for lesion preparation is to have adequate lesion preparation and controlled dissection to facilitate homogenous drug delivery. We also discuss the role of intravascular imaging and physiologic assessment-guided DCB intervention.

### 5.1. Lesion Preparation

**Figure 1:**
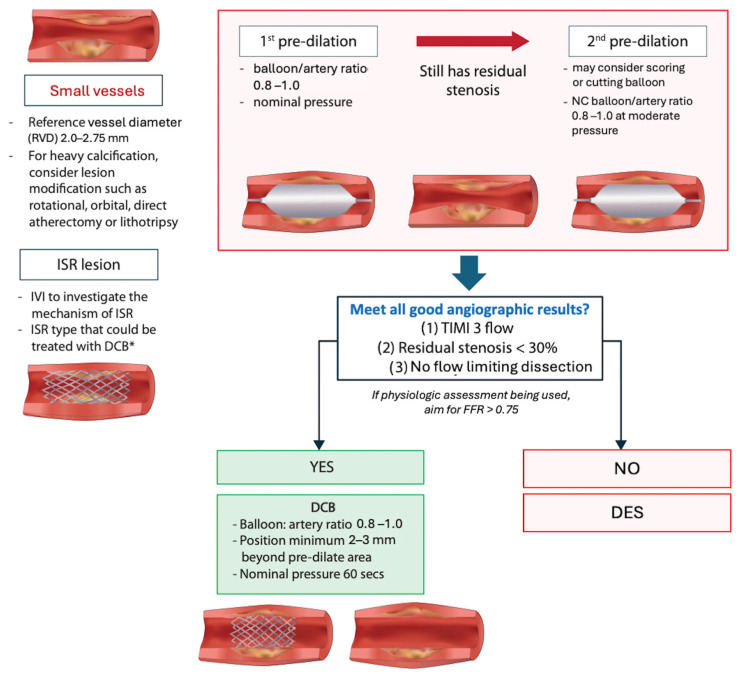
Overview of DCB treatment in ISR lesion and de novo lesion. * Non-type I ISR.

Pre-dilation prior to DCB deployment is recommended with a balloon-to-vessel size ratio of 0.8–1.0 and the inflation pressure higher than the nominal pressure [112]. Figure 1 shows the overview of DCB treatment in ISR lesions and de novo lesions. The type of pre-dilation balloon used depends on the complexity of the lesions. For complex lesions, it is advisable to consider non-compliant balloons with high pressure or scoring balloons to optimize lesion preparation. Several studies support the use of scoring balloons over conventional balloons to prepare lesions suitable for drug-coated balloon angioplasty, either ISR [113] or de novo lesions [114]. The scoring balloon leads to a significant reduction in binary restenosis [113] and target lesion failure (TLF) [114]. In the calcified lesion, plaque modification devices such as rotational/orbital atherectomy and intravascular lithotripsy should be considered and reassessed, as well as the feasibility of the use of DCBs after lesion preparation. CIAT statement for the lesion preparation as shown in Box 8.

Box 8 **CIAT statement for the lesion preparation before DCB treatment.**

Statement 8: A balloon-to-vessel ratio of 0.8–1.0 and inflation pressure above nominal are recommended. The choice of balloon should be tailored to lesion complexity to minimize residual stenosis and optimize drug uptake.

- Level of Evidence: Moderate.
- Level of consensus: 93.3% Agree; 6.7% Neutral; 0% Disagree.

**Figure 2:**
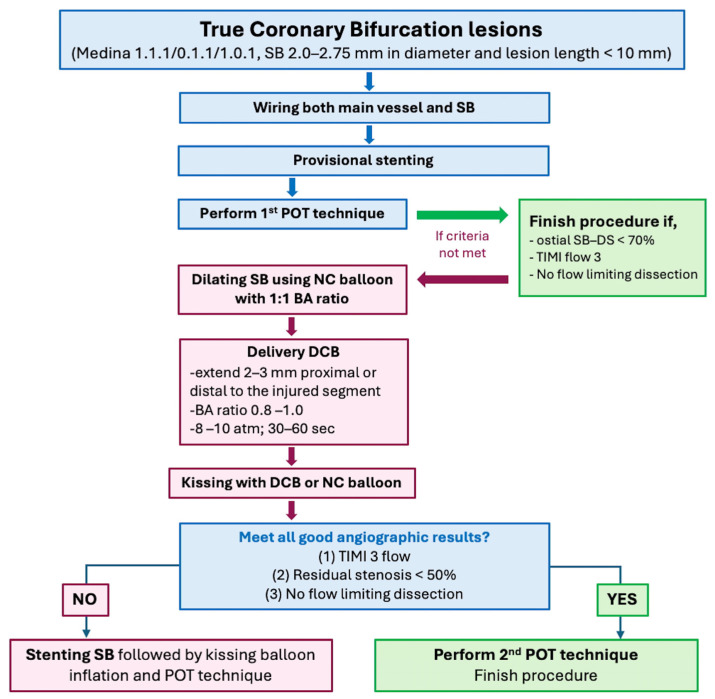
Overview of DCB treatment in bifurcation lesions. Abbreviation: DCB, drug-coated ballon; DS, diameter stenosis; NC, non-compliant balloon; POT, proximal optimization therapy; SB, side branch.

For lesion preparation in bifurcation lesions, the protocol of the DCB-BIF trial can be followed [72]. After provisional stenting and performing POT [115], if the ostial SB stenosis is ≥70%, the SB should be dilated using NC balloons with a balloon-to-artery ratio of 1:1, then followed by the DCB with the same balloon-to-artery ratio. The study protocol mandates that the final kissing balloon technique be performed using NC balloons in the SB rather than the DCB itself. However, in practice, some operators use the DCB for the final kissing balloon technique to save procedural time. It remains unclear whether one method offers any advantages over the other. Figure 2 shows the overview of using DCBs for side branch treatment.

### 5.2. How to Define That the Lesion Is Adequately Pre-Dilate?

The follow-up angiogram should include the following checklist as shown in Figure 1: (1) TIMI flow grade 3; (2) residual stenosis ≤ 30%; and (3) no flow-limiting dissection. If all these criteria are met, DCB treatment can proceed. However, if the stenosis remains, repeat prolonged balloon inflation (30–60 s) at nominal pressure with an NC balloon or specialty balloons such as scoring and cutting balloons with a 0.8–1:1 balloon-to-artery diameter ratio should be considered. In case of resistant lesions, gradual inflation of a high-pressure noncompliant balloon or specialty balloons can be used, but the operator may downsize the balloon by 0.5 mm to prevent potential complications from high-pressure inflation [116].

The rate of coronary dissection after lesion preparation from coronary angiography is approximately 14.7–39.1% [117,118,119], while the dissection rate can be as high as 97.2% from optical coherence tomography [118]. Bailout stent implantation due to dissection occurs in about 11.9–21.1% of cases [117,119]. Despite the common occurrence of coronary dissection after DCBs, the dissection does not seem to correlate with the adverse events during midterm follow-up [119]. This suggests that further research is needed to determine the severity of dissection that is considered safe and does not lead to future complications. CIAT statement for lesion assessment before DCB treatment as shown in Box 9.

Box 9 **CIAT statement for lesion assessment before DCB treatment.**

Statement 9: Angiographic confirmation of adequate lesion preparation is mandatory before DCB angioplasty. The lesion is considered suitable for DCB only when all criteria are met: (1) TIMI flow grade 3; (2) residual stenosis ≤ 30%; and (3) no flow-limiting dissection.

- Level of Evidence: Moderate.
- Level of consensus: 93.3% Agree; 6.7% Neutral; 0% Disagree.

### 5.3. Delivery of Balloon

The coated drug is designed to dissolve immediately after contact with blood. The diameter of the DCB is important, and the balloon-to-artery ratio should be 1:1 since the small DCB-to-stent ratio (≤0.91) in the ISR lesion is one of the predictors of TLF [120]. To avoid losing effective medication, it was recommended that the DCBs be delivered to the target lesion ≤ 45 s [118]. The longer device transit time could lead to a lower arterial drug retention after delivery [121]. If the lesion is in tortuous vessels or distal to a calcified lesion, an extension catheter is highly recommended, as using a pre-dilation balloon does not guarantee successful DCB delivery.

The DCB should be positioned to land at least 2–3 mm proximal and distal to the lesion to avoid geographical mismatch. Additionally, the balloon should be inflated at nominal pressure for a minimum of 60 s, as inflation times shorter than 60 s have been associated with TLF [120].

In terms of inflation pressure of the DCB, a comparison was made between oversized DCB inflation at low pressure (<4 atm, 2.4 ± 1.2 atm, DCB/artery ratio 1.14 ± 0.22; LP group) and 135 lesions (119 patients) treated with the standard DCB technique (7.1 ± 2.2 atm, DCB/artery ratio 1.03 ± 0.16; SP group) [122]. The findings indicated that there were no significant differences in the National Heart, Lung, and Blood Institute (NHLBI) types of dissections or late restenosis between the oversized low-pressure technique and the standard technique [122]. Additionally, a study on porcine carotid arteries compared DCB-to-artery ratios of 1:1 and 1.25:1, revealing that a higher DCB-to-artery ratio resulted in increased arterial drug concentrations with shorter transit times [121].

To detect acute vessel recoil after using DCBs, a final assessment is recommended at least 5 min after DCB angioplasty and a bolus of intracoronary vasodilator [21,123]. In this scenario, bailout stent implantation should be considered [21]. CIAT statement for optimal DCB delivery as shown in Box 10.

Box 10 **CIAT statement for optimal DCB delivery.**

Statement 10: For optimal drug transfer and procedural success, DCB should be delivered to the target lesion within 45 s, inflated for at least 60 s and assessed for acute recoil after delivery at least 5 min.

- Level of Evidence: Moderate.
- Level of consensus: 100% Agree; 0% Neutral; 0% Disagree.

### 5.4. Role of Coronary Physiology-Guided DCB Treatment

It has been reported that DCB treatment after lesion preparation with a fractional flow reserve (FFR) value ≥ 0.75 was safe and associated with a lower rate of restenosis [112]. However, it is important to note that there is a discordance between residual diameter stenosis and the FFR values [124]. Approximately 68.7% of patients exhibited residual stenosis > 30% despite having FFR ≥ 0.75, while 7.1% showed a reverse mismatch. Moreover, the immediate blood flow after DCB treatment differs from that of DESs. In the case of the DES, the stent area directly reflects the immediate gain of the flow, whereas in the DCB, the flow area has the potential to increase over time due to late remodeling.

The FADDY (Fractional Flow Reserve–Guided Drug-Coated Balloon Only Strategy in De Novo Coronary Lesions) trial hypothesized the noninferiority of DCBs compared to DESs in terms of FFR at the 9-month follow-up. A significant difference in FFR was immediately observed after coronary revascularization between the two arms (0.88 vs. 0.91; *p* < 0.001). However, there were no significant differences in FFR at the 9-month follow-up. The late lumen loss at the 9-month follow-up was −0.01 mm in the DCB arm, whereas it was 0.12 mm in the DES arm (*p* = 0.03) [125].

Yamamoto and colleagues conducted serial physiological assessments, specifically measuring FFR and instantaneous wave-free ratio (iFR) at 0 and 15 min after DCB inflation. In patients who developed LLE at 9 months, FFR and iFR at 15 min were significantly higher compared to the non-LLE group. The FFR values were 0.90 ± 0.03 for the LLE group versus 0.85 ± 0.07 for the non-LLE group (*p* < 0.001). Similarly, the iFR values were 0.97 ± 0.02 compared to 0.92 ± 0.10 (*p* = 0.008) [126]. Notably, the type of dissection that occurred after balloon angioplasty (BA) was found not to correlate with residual FFR.

Angiographic-derived fractional flow reserve (FFR) is a novel, non-invasive method that estimates FFR using three-dimensional quantitative coronary angiography (QCA) without the need for hyperemia or pressure guidewires. It provides both anatomical and functional assessments of coronary lesions, which can be crucial in guiding DCB intervention. A retrospective analysis of AccuFFRangio V1.0 (ArteryFlow Technology, Hangzhou, China) following DCB procedures determined that a value of post-AccuFFR ≤ 0.87 is the optimal cutoff for predicting major adverse cardiac events (MACE), with an area under the curve (AUC) of 0.87 [127]. The events were driven by the repeat target vessel revascularization. Other platforms of angiographic-derived FFR, such as Murray law-based quantitative flow ratio (μQFR) [75] and quantitative flow ratio (QFR) [128], were also studied on the optimal cut-off values to predict vessel-oriented composite endpoint (VOCE) for DCB treatment. The best cut-off post-procedural μQFR and QFR is ≤0.89 [75,128]. A recent QUADRIC study reported that patients with post-PCI QFR < 0.90 had significantly higher rates of MACEs (~12.6%) compared to those with QFR ≥ 0.90 (~4.5%, *p* = 0.011) over two years [129]. Besides the post-procedure angiographic-derived FFR absolute value, the post-procedural QFR gradient across the lesion was also the predictor of TLR; the best cutoff value of post-procedural QFR gradient for predicting TLR was 0.08 [130].

Recently, in the TRANSFORM I study, patients treated with SCB had lower mean QFRs following the treatments compared to patients treated with PCBs (0.86 ± 0.15 vs. 0.91 ± 0.09, *p* = 0.026) and more vessels with a QFR ≤ 0.80. Patients in the SCB group had a higher proportion of binary stenosis than the PCB group (32.8% vs. 12.5%, odd ratio 3.41; 95% CI: 1.36–9.44; *p* = 0.012) [118]. The angio-derived FFR may serve as a useful alternative to assess whether lesion preparation is adequate before deploying a DCB.

#### When and How to Apply Physiology on DCB Treatment?

The application of physiologic assessment in the DCB treatment can be used in the following three aspects:

(1) Pre-procedural planning: The longitudinal pullback analysis of pressure wire or angiographic-derived FFR helps define lesion patterns and guide treatment decisions. Lesions with diffuse disease may benefit from DCB treatment from the possibility of late positive remodeling. DCBs should be considered only if FFR ≥ 0.75 after lesion preparation [131].
(2) Improving the precision of PCI: Physiologic assessment can identify the location of the residual target flow-limiting disease, ensure successful lesion preparation, and detect complications before DCB intervention.
(3) Post-procedural assessment: Post-PCI physiological assessment, especially QFR < 0.90, is associated with increased MACE risk. Serial QFR assessment after lesion preparation may aid in guiding final treatment and predicting prognosis.

### 5.5. Role of Intravascular Imaging-Guided DCB Treatment

In the early stage of DCB use, procedural success was primarily assessed using angiographic criteria. However, it is now well established that intravascular imaging (IVI)-guided PCI, particularly with DES implantation, leads to improved clinical outcomes [132]. IVI techniques, including intravascular ultrasound (IVUS) and optical coherence tomography (OCT), are superior to angiography in assessing luminal dimensions, vessel diameter, and detecting stent underexpansion, malapposition, tissue protrusion, stent edge dissections, and plaque burden at the stent edge that can be missed by angiography [133].

IVI provides high-resolution tomographic images that offer detailed information on coronary anatomy, which is essential for identifying the type and underlying mechanism of ISR and for tailoring treatment strategies [134]. It is reasonable to use IVI-guided DCB procedures, as they could potentially yield similarly favorable outcomes as seen with DESs.

The success of DCB therapy is highly dependent on optimal lesion preparation. An inadequate lesion preparation could compromise procedural outcomes. In the pre-PCI phase, IVI helps assess lesion morphology, determine the need for plaque modification [135] (e.g., atherectomy, NC balloon/scoring balloon/cutting balloon/intravascular lithotripsy), and appropriately size devices. DCB sizing by using the mean reference lumen diameter from IVI (1:1) is recommended.

**Figure 3:**
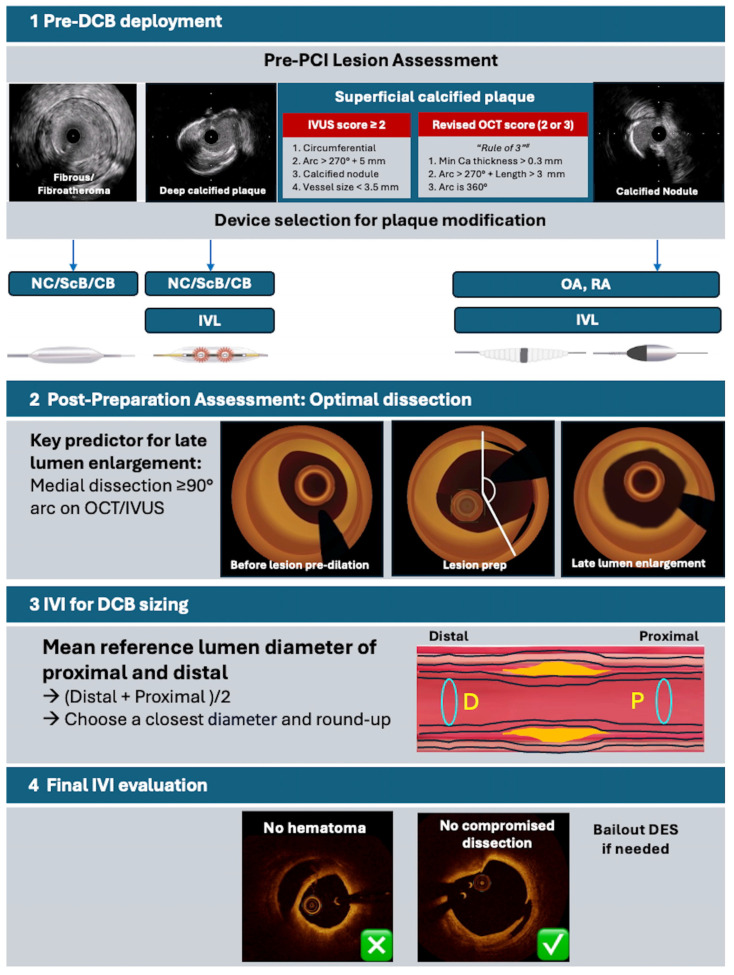
Intravascular imaging-guided DCB treatment. Abbreviation: CB, cutting balloon; DCB, drug-coated ballon; DES, drug-eluting stent; IVI, intravascular imaging; IVL, intravascular lithotripsy; IVUS, intravascular ultrasound; NC, non-compliant balloon; OA, orbital atherectomy; OCT, optical coherence tomography; POT, proximal optimization therapy; RA, rotational atherectomy; ScB, scoring balloon; SB, side branch.

After lesion preparation, IVI assists in evaluating dissection extent, sizing the DCB, and planning the treatment zone. After DCB deployment, IVI evaluates treatment effectiveness, the type and extent of dissections, and whether a bailout DES is needed [136]. Figure 3 summarizes IVI-guided DCB treatment.

The ULTIMATE-III trial (Intravascular Ultrasound vs. Angiography-Guided Drug-Coated Balloon Angioplasty) evaluated IVUS-guided DCB use in patients at high bleeding risk and de novo coronary lesions. Compared to angiography-guided procedures, IVUS guidance was associated with significantly lower late lumen loss, a larger minimal lumen diameter, and a lesser percent diameter stenosis. Although target vessel failure rates at 6 months were numerically lower with IVUS (0.8% vs. 3.1%), the difference did not reach statistical significance [137].

The Transform I [118] trial compared SCBs with PCBs in small vessel de novo lesions (≤2.75 mm). The SCB failed to demonstrate non-inferiority for net lumen gain at 6 months. Importantly, two different OCT-based balloon sizing strategies were used: SCB sizing was based on the distal reference diameter or rounded up no more than 0.25 mm, while PCB sizing used the mean diameter of proximal and distal references [138]. These differences in sizing methodology may have contributed to SCBs not meeting the non-inferiority endpoint.

IVUS has also shed light on LLE, a phenomenon observed following PCB angioplasty. Serial IVUS studies have demonstrated that LLE is characterized by both vessel enlargement (increased EEL area) and plaque volume regression. The “dissection index”, calculated as the sum of dissection points at a 1 mm cross-section divided by lesion length, emerged as the strongest predictor of LLE. Dissections may enhance drug penetration into the adventitia, thus promoting LLE [139].

OCT studies following PCB angioplasty have further detailed mechanisms of LLE, including vessel expansion, dissection flap healing, and plaque regression. Predictors of LLE included thick-cap fibroatheroma and post-procedure deep dissection reaching the tunica media [140].

Another OCT-based study identified layered plaque morphology and medial dissection with an arc > 90° as independent predictors of LLE. Although the exact relationship between layered plaques and LLE is not fully understood, LLE was associated with a lower rate of binary restenosis and repeat revascularization [141]. Conversely, absence of post-PCI medial dissection on OCT was associated with higher rates of TLF after DCBs in de novo lesions [142].

Despite these findings, unlike DES procedures, there are currently no established IVI-based criteria defining the “optimal result” for DCB angioplasty—such as acceptable residual stenosis, luminal gain, or dissection extent. Further studies are warranted to define these procedural targets and to optimize DCB outcomes using IVI guidance. CIAT statement for the use of intravascular imaging guided DCB treatment as shown in Box 11.

Box 11 **CIAT statement for the use of intravascular imaging guided DCB treatment.**

Statement 11: Integration of intravascular imaging into DCB procedures enhances procedural precision and is recommended, particularly for complex, calcified, or restenotic lesions.

- Level of Evidence: Moderate.
- Level of consensus: 100% Agree; 0% Neutral; 0% Disagree.

Statement 12: Presence of medial dissection on intravascular imaging before DCB inflation is recommended to achieve favorable angiographic and clinical outcomes.

- Level of Evidence: Moderate.
- Level of consensus: 80% Agree; 20% Neutral; 0% Disagree.

### 5.6. Hybrid Strategy

Implantation of long metallic stents in de novo diffuse CAD is associated with suboptimal clinical outcomes, including TLF, cardiac death, TLR, and stent thrombosis [143]. To avoid full-metal jacket implantation, alternative strategies such as DCB-only angioplasty or a hybrid approach that combines DCBs with DESs have emerged.

The main concept behind the hybrid strategy is to minimize the total stent length in complex coronary lesions. This is clinically relevant because stent length is an independent predictor of in-stent restenosis (ISR) and stent thrombosis. Moreover, the presence of a “metallic cage” may impair coronary vasomotion and limit future options for coronary artery bypass grafting (CABG) [144].

**Figure 4:**
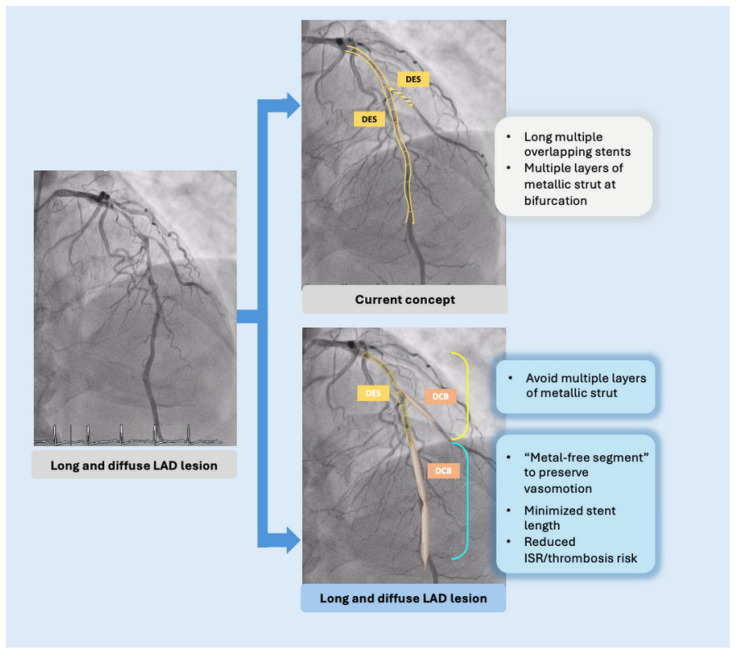
Hybrid strategies for diffuse CAD and bifurcation lesions.

Theoretically, DCB-only treatment would be ideal for treating long, diffuse disease. However, in cases of highly complex lesions, complications such as severe coronary dissection after balloon dilation or significant residual stenosis can occur. In these situations, a hybrid approach that combines both DESs and DCBs may provide a more pragmatic solution. Figure 4 demonstrates the hybrid approach for diffuse CAD and bifurcation lesions.

In a study by Costopoulos et al. [34], patients with diffuse CAD were treated with PCBs, and outcomes were compared with the DES cohort. A DCB-only strategy was used in 56.0% of cases, bailout DES in 7.4%, and a hybrid strategy in 36.6%. At the 2-year follow-up, the outcomes of DCBs ± DESs were comparable to DESs alone. Notably, the lesion length treated in the hybrid group (67.7 ± 13.4 mm) was significantly longer than that in the DCB-only group (35.4 ± 5.7 mm), highlighting the hybrid strategy’s capacity to address more complex anatomy.

Conversely, a retrospective study evaluating DCB angioplasty for de novo long lesions on large coronary vessels (>3 mm), predominantly using SCBs (77%), reported a 12-month rate of 5.1%. This included 1.5% in the DCB-only group versus 10.7% in the hybrid group (*p* = 0.073) [145], suggesting a trend toward increased events in the hybrid arm, although not statistically significant.

In a separate case series assessing the functional outcomes of the hybrid strategy, the mean post-PCI QFR value was 0.90 ± 0.10. When procedural success was defined as QFR > 0.80 in any treated vessel, 96.3% of patients achieved it [146], supporting the strategy’s efficacy in preserving physiological outcomes.

The ongoing HYPER Pilot Study (NCT03939468) is a prospective, observational trial evaluating the safety and effectiveness of the hybrid approach in de novo diffuse CAD. In this study, a hybrid strategy is defined as overlapping or slightly (2–3 mm) superimposing a new generation DES implantation in the proximal, larger vessel segment, combined with DCB angioplasty in a distal small vessel segment or at a bifurcation side branch. The primary endpoint is the device-oriented composite endpoint (DOCE) of cardiac death, target vessel myocardial infarction (TVMI), and ischemia-driven target lesion revascularization (ID-TLR) at 12 months [147]. CIAT statement for the hybrid strategy as shown in Box 12.

Box 12 **CIAT statement for the hybrid strategy.**

Statement 13: A hybrid strategy combining DES implantation (proximal segment of the lesion) and DCB angioplasty (distal segment or side branch) is a practical and effective alternative for treating de novo diffuse CAD or bifurcation.

- Level of Evidence: Moderate.
- Level of consensus: 86.7% Agree; 13.3% Neutral; 0% Disagree.

## 6. Antiplatelet Therapy After DCB-Only PCI

Dual antiplatelet therapy (DAPT) is the standard of care following PCI, with recommendations typically ranging from 6 to 12 months [148]. This strategy primarily aims at preventing stent thrombosis and restenosis associated with DES implantation. However, in patients undergoing DCB-only angioplasty, where no permanent metallic scaffold is implanted, the risk of vessel thrombosis is substantially lower, potentially allowing for shorter durations of DAPT [38].

To date, RCTs specifically comparing different antiplatelet regimens after DCB-only PCI are limited. Consequently, evidence regarding DAPT duration after DCBs is largely derived from RCTs comparing DCBs to DESs or from prospective observational studies evaluating DCBs across diverse clinical scenarios.

**Figure 5:**
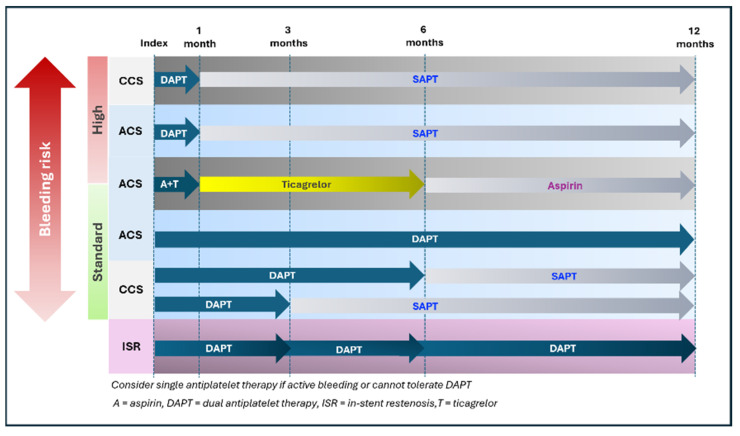
Duration of dual antiplatelet therapy following DCB-only PCI.

The optimal DAPT duration after DCB-only PCI should be individualized, considering the clinical presentation (e.g., chronic vs. acute coronary syndrome), bleeding and thrombotic risks, and lesion characteristics (e.g., de novo vs. in-stent restenosis). Nevertheless, upon completion of DAPT, lifelong single antiplatelet therapy (SAPT) is advised. Figure 5 summarizes the optimal duration for antiplatelet after DCB-only PCI.

In patients at high bleeding risk (HBR) with non-complex de novo coronary lesions, DAPT may be safely shortened to 1 month. This approach is supported by RCT data demonstrating the superiority of DCBs over bare-metal stents with a 1-month DAPT regimen in HBR populations [23].

For patients with standard bleeding risk, most expert consensus guidelines recommended a DAPT duration of 3 to 6 months following DCB-only PCI for CCS and up to 12 months in the setting of ACS. An alternative DAPT regimen in ACS patients is the stepwise de-escalation strategy that combines aspirin and ticagrelor for the first month, followed by ticagrelor monotherapy for 5 months, and then aspirin monotherapy thereafter. This regimen showed non-inferiority to the standard 12-month DAPT approach in the REC-CAGRFREE II study [149]. Notably, around 20.6% of the study population were classified as HBR, suggesting this strategy may also be applicable in that subgroup.

Regarding DAPT duration after DCB treatment of ISR, previous studies have used durations ranging from 3 to 12 months. However, no dedicated trials have specifically evaluated the optimal DAPT duration following DCB angioplasty in ISR lesions.

In cases where patients are unable to tolerate DAPT or experience active bleeding during treatment, early transition to single antiplatelet therapy (SAPT) may be considered following a careful assessment of ischemic versus bleeding risk. Observational data suggest that SAPT may be a safe and feasible option in selected cases under these circumstances [150,151].

Evidence on optimal antiplatelet strategies following DCB-only PCI in patients requiring long-term oral anticoagulant therapy remains limited, as most landmark studies in this population have focused on DES-treated patients [152]. Until further data are available, clinical decisions in this subgroup should be made on an individual basis, balancing thrombotic and hemorrhagic risks.

In cases where DCBs are used in conjunction with DESs, either by design (hybrid strategy) or due to bailout stenting, the duration of DAPT should follow current guideline recommendations for DES implantation. CIAT statement for the duration of DAPT after DCB treatment as shown in Box 13.

Box 13 **CIAT statement for the duration of DAPT after DCB treatment.**

Statement 14: DAPT duration should reflect clinical and bleeding risk: 1 month for HBR, 3–6 months for CCS, and up to 12 months for ACS, followed by lifelong SAPT.

- Level of Evidence: Moderate.
- Level of consensus: 93.3% Agree; 6.7% Neutral; 0% Disagree.

Statement 15: When DCBs are combined with DESs, follow standard DES DAPT guidance.

- Level of Evidence: Moderate.
- Level of consensus: 93.3% Agree; 6.7% Neutral; 0% Disagree.

## 7. Conclusions

DCBs have become an essential tool in the treatment of various coronary artery lesions, offering a viable alternative or complement to DESs. Evidence supports their use in complex settings such as ISR, small vessel disease, bifurcation lesions, and calcified lesions. Optimal lesion preparation, careful procedural techniques, and integration of intravascular imaging and physiological assessment are critical to maximizing the benefits of DCB therapy. In Thailand, the adoption of DCBs under national reimbursement guidelines reflects their growing clinical importance. This consensus statement aims to support clinicians in using DCB technology.

## Figures and Tables

**Table 1:** Summary of clinical trials comparing drug-coated balloons and drug-eluting stents or balloon angioplasty in the in-stent restenosis setting.

Author (Year)	Number of Patients/Clinical Trials	Comparable Groups	Principle Findings
Cai J-Z et al. [13] (2017)	913 patients/5 trials	New-generation DES vs. DCB	New-generation DESs were significantly Higher in acute luminal gain (−0.31 mm, 95% CI [−0.42 to −0.20], *p* < 0.001). Lower in percent diameter stenosis (RR 0.28, 95% CI [0.02 to 0.55], *p* = 0.04). Lower in TLR (RR: 1.96, 95% CI [1.17 to 3.28], *p* = 0.01) No statistical differences in MACEs (RR 1.21, 95% [CI 0.67 to 2.17], *p* = 0.53). MI (RR 1.16, 95% CI [0.55 to 2.48], *p* = 0.69). Cardiac death (RR 1.80, 95% CI [0.60 to 5.39], *p* = 0.29).
Zhu Y et al. [14] (2021)	1193 patients/5 trials	DES vs. DCB	DES was associated with a significant reduction in the term of TLR (RR 1.53, 95% CI [1.15 to 2.04], *p* = 0.003). But no significant difference in Angiographic outcomes. Other clinical endpoints (MACE, death, MI). EES was associated with a Lower TLR (RR 2.43, 95% CI [1.28 to 4.62], *p* = 0.007). Larger minimum lumen diameter (MD −0.25, 95%CI [−0.38 to −0.11], *p* = 0.000). Lower percent diameter stenosis (MD 7.29%, 95% CI [2.86 to 11.71%], *p* = 0.001). Less binary restenosis (OR 2.20, 95% CI [1.18 to 4.11], *p* = 0.01).
Sabina M et al. [15] (2024)	1322 patients/6 trials	DCB vs. POBA	DCBs significantly reduced Late in-stent and in-segment luminal loss (*p* < 0.001). Target lesion revascularization (*p* = 0.02). MACEs (*p* < 0.00001). The combined endpoint of TLR, MI, and death (*p* = 0.0002). No significant differences in MI. Mortality rates.
Abdelaziz A et al. [16] (2024)	1977 patients/10 trials	DES vs. DCB	DCB significantly increased TLR (OR 1.54, 95% CI [1.2 to 1.99]). No significant difference in MACEs (OR 1.04, 95% CI [0.87 to 1.44]). LLL (MD −0.08, 95% CI [−0.18 to 0.02]).
Shaikh S et al. [17] (2024)	1171 patients/6 trials	DCB vs. POBA	DCB significantly decreased TVR (OR 0.33, 95% CI [0.19 to 0.57]. TVF (OR 0.30, 95% CI [0.09 to 0.99]). TLR (OR 0.22, 95% CI [0.10 to 0.46]). Restenosis (OR 0.1343, 95% CI [0.06 to 0.27]). MACEs (OR 0.2, 95% CI [0.12 to 0.37]). No significant difference in MI (OR 0.6, 95%CI [0.0349 to 1.07]). All-cause mortality (OR 0.8, 95% CI [0.363 to 2.09]).
Al-Abdouh A et al. [18] (2024)	1343 patients/6 trials	PCB vs. POBA	PCB significantly decreased TLR (RR 0.28, 95%CI [0.11 to 0.68]). MACE (RR 0.35, 95%CI [0.20 to 0.64]). No significant difference in All-cause mortality (RR 0.56, 95%CI [0.14 to 2.31]). CV mortality (RR 0.61, 95% CI [0.02 to 16.85]). MI (RR 0.60, 95%CI [0.27 to 1.31]). Stent thrombosis (RR 0.13, 95%CI [0.00 to 5.06]).
Oliveira VMR et al. [19] (2025)	1349 patients/7 trials	PCB vs. POBA	PCB significantly lower in TLR (RR 0.31, 95% CI [0.18 to 0.52]; *p* < 0.01). TVR (RR 0.53, 95% CI [0.42 to 0.67]; *p* < 0.01). MACEs (RR 0.25, 95% CI [16 to 0.38]; *p* < 0.01). MI (RR 0.59, 95% CI [0.37 to 0.95]; *p* = 0.03). No significant differences in All-cause mortality (RR 0.79, 95% CI 0.37 to 1.70]; *p* = 0.54). Cardiac death (RR 0.46, 95% CI 0.03 to 8.12]; *p* = 0.60). TLF (RR 0.39, 95% CI [0.13–1.11]; *p* = 0.08). Stent thrombosis (RR 0.21, 95% CI [0.03–1.35]; *p* = 0.10).

Abbreviations: CI, confidence interval; CV, cardiovascular; DCB, drug-coated balloon; DES, drug-eluting stent; EES, everolimus-eluting stent; LLL, late lumen loss; MACEs, major adverse cardiac events; MD, mean difference; MI, myocardial infarction; mm, millimeter; OR, odd ratio; POBA, plain balloon angioplasty; PCB, paclitaxel-coated balloon; RR, risk ratio; TLF, target lesion failure; TLR, target lesion revascularization; TVR, target vessel revascularization.

**Table 2:** Randomized controlled trials of DCBs in de novo lesions of small coronary vessels.

Study (Year)	Comparators	N	Follow-Up Duration	Angiographic Follow-Up	MACE (%); Relative Risk	Target Vessel Thrombosis (%); Relative Risk	MI (%); Relative Risk	TLR (%)
Funatsu et al. [22] (2017)	DCB vs. POBA	135	6 mo (Clinical)	LLL (0.01 ± 0.31 vs. 0.32 ± 0.34 mm; *p* < 0.01)	NR	NR	3.4 vs. 10.3	2.3 vs. 10.3
DEBUT [23] (2019)	DCB vs. BMS	206	9 mo (Clinical)	NR	NR	0 vs. 2	1 vs. 14(%); RR = 0.07	NR
PICCOLETO [24] (2010)	DCB vs. PES	57	6 mo (angio) 12 mo (clinical)	LLL 0.13 mm (−0.14 to 0.57 mm) vs. 0.10 mm (−0.16 to 0.34 mm)	13.9 vs. 14.1 (%); RR = 0.98	0.5 vs. 1.6 (%); RR = 0.33	5 vs. 6.1 (%); RR = 0.81	NR
BELLO [26] (2012)	DCB vs. PES	182	6 mo (Angio) 12 mo (Clinical)	MLD 1.11 ± 0.65 mm vs. 1.94 ± 0.72 mm; *p* = 0.0002	9.3 vs. 18.4 (%); RR = 0.51	0 vs. 3.5 (%); RR = 0.11	1.7 vs. 6.1 (%); RR = 0.28	7.6 vs. 13.2
RESTORE SVD [28] (2018)	DCB vs. ZES	230	9–12 mo (Angio) 12 mo (Clinical)	diameter stenosis 29.6 ± 2.0% vs. 24.1 ± 2.0%; *p* < 0.001)	6 vs. 5.3 (%); RR = 0.15	NR	NR	6. vs. 2.6
BASKET-SMALL 2 [30] (2012)	DCB vs. PES/EES	758	6 mo (Angio) 12 mo (Clinical)	LLL 0.08 ± 0.38 mm vs. 0.29 ± 0.44 mm; *p* = 0.001	14.4 vs. 30.4 (%); RR = 0.47	0 vs. 0 (%); RR = 1.02	8.9 vs. 18.5 (%); RR = 0.48	6.7 vs. 13.0

Abbreviations: as shown in Table 1. BMS, bare-metal stent; MI, myocardial infarction; MLD, minimal lumen diameter; NR, not report; PES, paclitaxel-eluting stent; ZES, zotarolimus-eluting stent.

**Table 3:** Summary of clinical studies on the use of drug-coated balloons for calcified lesions.

Authors (Year)	Number (Patients /PCI)	Study Design	Plaque Modification Technique	Comparable	Type of DCB	Follow-Up Period	Outcomes
Rissanen T et al. [29] (2017)	65/82	Retrospective single center	RA	Single arm	PCB	17 months (median)	MACEs (the composite of CV death, ischemia-driven TLR, or non-fatal MI 14% at 12 months. 20% at 24 months. The rate of ischemia-driven TLR 1.5% at 12 months 3.0% at 24 months.
Ueno K et al. [50] (2019)	123/166	Retrospective single center	RA	DCB vs. DES	PCB	732 days (median)	No significant difference (after propensity score analysis) (DCB vs. DES) TLR (12.9% vs. 16.3%; *p* = 0.70). TVR (12.9% vs. 26.1%; *p* = 0.17).
Iwasaki Y et al. [51] (2021)	157/184	Retrospective single center	RA	DCB vs. DES	NA	1 year	No significant difference (DES vs. DCB) MACE (8% vs. 11%; *p* = 0.30). Cardiac death (0% vs. 0%). Noncardiac death (4% vs. 3%; *p* = 0.36). Target-vessel-related MI (0% vs. 0%). TLR (4% vs. 8%; *p* = 0.30). Major bleeding (1% vs. 0%).
Mitsui K et al. [52] (2023)	135 patients	Retrospective single center	OA	DCB vs. DES	PCB	1 year	No significant difference in 1-year MACE free rate (90.3% in DCB vs. 96.6% in DES; *p* = 0.136).
Shan Y et al. [48] (2023)	1263/1392	Retrospective, 3 centers	RA (11.6% of the calcified group)	Calcified vs. non-calcified lesion	PCB	3 years	Significant differences after propensity score matching (calcified vs. non-calcified) TLF (9.52% vs. 4.94%, OR 2.080, 95% CI [1.083 to 3.998]; *p* = 0.034). TLR (7.41% vs. 2.88%, OR 2.642, 95% CI [1.206 to 5.787]; *p* = 0.020). Non-significant difference after propensity score matching (calcified vs. non-calcified) MACE (12.35% vs. 7.82%, OR 1.665, 95% CI [0.951 to 2.916]; *p* = 0.079). Cardiac death (2.06% vs. 2.06%, OR 0.995, 95% CI [0.288 to 3.436]; *p* = 0.993). MI (1.23% vs. 0.82%, OR 2.505, 95% CI [0.261 to 8.689]; *p* = 0.652). Any revascularization (12.76% vs. 9.67%, OR 1.256, 95% CI [0.747 to 2.111]; *p* = 0.738).
Dong H et al. [53] (2023)	318/322	Retrospective single center	RA	DCB vs. DES	PCB	15 months (DCB) 22 months (DES) (median)	Non-significant difference (DES/DCB) MACCE (18.77% vs. 12.28%; *p* = 0.244). All-cause mortality (1.53% vs. 0%; *p* = 0.776). TLR (13.79% vs. 7.02%; *p* = 0.239). MI (1.15% vs. 0%; *p* = 1.00). Stroke (2.29% vs. 3.51%; *p* = 0.951).

Abbreviation: CI, confidence interval; CV, cardiovascular; DCB, drug-coated balloon; DES, drug-eluting stent; MI, myocardial infarction; PCB, paclitaxel-coated balloon; PCI, percutaneous coronary intervention; RA, rotational atherectomy; MACE, major adverse cardiac event; MACCE, major adverse cardiovascular and cerebrovascular event; NA, not available; OA, orbital atherectomy; OR, odd ratio; TLF, target lesion failure; TLR, target lesion revascularization; TVR, target vessel revascularization.

**Table 4:** Summary of clinical data on the use of DCBs in CTO lesion.

Author (Year)	Patients /Lesions	Follow-Up	MACE	MI	TLR	TVR	Cardiac Death
Koln PJ et al. [55] (2016)	34/66	8.62 months (mean)	17.6%	0%	11.8%	7.4%	0%
Jun EJ et al. [56] (2022)	84/93	720 days (median)	8.3% (1 year) 16.7% (2 year)	0% (1 year) 3.6% (2 year)	NA NA	7.1% (1 year) 13.1% (2 year)	1.2% (1 year) 2.4% (2 year)
Terashita K et al. [57] (2023)	105/124	29 months (median)	NA	0%	8%	NA	0%
Wang X et al. [58] (2023)	591/290	3 years	11.4%	0.7%	6.0%	8.2%	2.1%

Abbreviation: as shown in Table 1 and Table 2.

**Table 5:** Summary of DCB platforms available in Thailand.

	Company	Excipient	Dose (µg/mm^2^)	Technique Used	Trial	CE	FDA
**Paclitaxel**							
Agent	Boston	AcetylTributyl Citrate (ATBC)	2	Proprietary coating	AGENT IDE [111]	Y	Y
Sequent please Neo	B.Braun	Iopromide	3	Developed with modified coating	BASKET SMALL-2 [25]	Y	NA
Pantera Lux	Biotronik	Butyryl tri-hexyl citrate (BTHC)	3	Proprietary hydrophilic non-polymeric carrier	PANTERA LUX [97]	Y	NA
Prevail	Medtronic	biocompatible urea	3.5	Open balloon Automate liquid formation	PREVAIL [98]	Y	NA
Swide	La Prima Medicare	Iopromide	3	Ultrasonic spraying	REC CAGE FREE-I [42]	NA	NA
**Sirolimus**							
Magic touch	Concept medical	Phospholipids based	1.27	Proprietary nanolute technology	EASTBOURNE [100]	Y	NA
Selution SLR	Cordis	MicroReservoirs embedded with Cell Adherent Technology (CAT coating)	1	proprietary amphipathic lipid technology	SIROOP [99]	Y	NA
**Biolimus**							
Bioascend	Biosensor	Polyethylene oxide (PEO)	3	PEO hydrophilic coating (Crystaline biolimus)	BIORISE [102]	Y	NA

Abbreviation: as shown in Table 1 and Table 2. CE, Conformité Européenne; FDA, U.S. Food and Drug Administration.

## Data Availability

No new data were created or analyzed in this study. Data sharing is not applicable to this article.
